# Makers of Man

**Published:** 1911-05

**Authors:** 


**Makers of Man.** By **Charles J. Whitby,** M.D., author of "The Logic of Human Character." Octavo, pp. 424. Illustrated. New York; Rebman Co. (Cloth, $3.00.)

This is a serious attempt to study the Great Men for the purpose of discovering if possible the physical and mental conditions which combine to make up their famous individualities.

He has chosen for the subjects of his experiment forty famous individuals, from the various ranks of human endeavor. Among the great administrators and warriors are Cesar, Charlemaigne, Lord Nelson and Napoleon; of the great rulers, Cromwell, Frederic the Great, Abraham Lincoln; of the great religious leaders, Christ, St. Paul, St. Augustine, Mahomet, Luther; of the scientists, Bacon, Galileo, Newton; of the musicians, Mozart and Beethoven; of the artists, Turner, Titian and Leonardo Da Vinci; of the philosophers, Renan, Emerson, Kant, Hegel, Leibnitz and Descartes; of the authors, Sir Walter Scott, Goethe, Dante and Flaubert.

Eugenics is the goal towards which our author is working. The study of masters of men, the makers of men, shows how impossible it is to assume that the great men come from any single type of the race, that none of them seem to represent any type of mental activity which is common to all, that above and beyond being great in every specialty, a man in order to be determined great must be not only a real artist but a great man; as the author says the mere artist is too naive and too ingenuous, that manliness is required as an attribute to greatness, for manliness is required of those who assume to govern the earth.

The book is most instructive in its careful study of the family history, parentage, constitution, of those selected for study and will yield much food for thought to those students of psychology who are seriously working to solve the problem of the improvement of the race and the knowledge of the forces which lead to eugenics. J. W. P.

